# GASS: genome structural annotation for Eukaryotes based on species similarity

**DOI:** 10.1186/s12864-015-1353-3

**Published:** 2015-03-04

**Authors:** Ying Wang, Lina Chen, Nianfeng Song, Xiaoye Lei

**Affiliations:** Department of Automation, School of Information Science and Technology, Xiamen University, Xiamen, Fujian 361005 China

**Keywords:** Structural genome annotation, Computational method, Species similarity, Dynamic programming, Rhesus genome

## Abstract

**Background:**

With the development of high-throughput sequencing techniques, more and more genomes were sequenced and assembled. However, annotating a genome’s structure rapidly and expressly remains challenging. Current eukaryotic genome annotations require various, abundant supporting data, such as: species-specific and cross-species protein sequences, ESTs, cDNA and RNA-Seq data. Collecting those data and merging their analytical results to achieve a consistent complete annotation is a complex, time and cost consuming task.

**Results:**

In our study, we proposed a fast and easy-to-use computational tool: GASS (Genome Annotation based on Species Similarity). It annotates a eukaryotic genome based on only the annotations from another similar species. With aligning the exons’ sequences of an annotated similar species to the un-annotated genome, GASS detects the optimal transcript annotations with a shortest-path model. In our study, GASS was used to achieve the rhesus annotations based on the human annotations. The produced annotations were evaluated by comparing them to the two existing rhesus annotation databases (RefSeq and Ensembl) directly and being aligned with three RNA-Seq data of rhesus. The experiment results showed that more than 65% RefSeq exons and splicing junctions were exactly found by GASS. GASS’s sensitivity was higher than RefSeq’s, and was close to Ensembl’s. GASS had higher specificities than Ensembl at gene, transcript, exon and splicing junction levels. We also found the mis-assemblies of rheMac3 genome, which led to the 2 bp shifts in annotating position on exons’ boundary and then the incomplete splicing canonical sites in Refseq annotations. These detections were further supported by various data sources.

**Conclusions:**

GASS quickly produces structural genome annotations in sufficient abundance and accuracy. With simple and rapid running of GASS, small labs can create quick views of genome annotations for an un-annotated species, without the necessity to create, collect, analyze and synthesize extra various data sources, or wait several months for the annotations from professional organizations. GASS can be applied to many study occasions, such as the analysis of RNA-Seq datasets from the unannotated species whose genome drafts are available but the annotations are not.

**Electronic supplementary material:**

The online version of this article (doi:10.1186/s12864-015-1353-3) contains supplementary material, which is available to authorized users.

## Background

With the development of high-throughput sequencing techniques, more and more genomes have been sequenced. Obtaining a high-quality draft assembly is an achievable goal for most genome projects. However, genome annotation remains a challenging task because of the difficulty to collect or pre-create the required ESTs, protein, RNA-Seq and other data sources; synthesize their results; train, optimize and configure gene annotation tools for exotic nature [1].

Traditional biological experiments can hardly tackle the whole genome structure annotations, which make computational methods indispensable. The existing computational approaches may be broadly classified into the following two categories [1]:

### Evidence alignment

The first approach is evidence alignment, which determines whether the region is transcribed and/or coded by exploiting a sufficient similarity between a genomic sequence region and protein sequences, ESTs, or RNA-Seq data [2]. RNA-Seq data has the greatest potential to improve the accuracy of genome structure annotations. Annotations with RNA-Seq data were generally handled in two ways: ① *de novo* transcript assembly, such as ABySS [3], SOAPdenovo [4] and Trinity [5]. One of the typical annotation pipeline is PASA [6]. ② aligning to genome and assembling the alignments, such as TopHat [7] and Cufflinks [8]. The typical annotation pipeline is MAKER [9]. However, RNA-Seq is not trivial to use because of their short length and large size [1]. For example, Trinity identified 54% of known mouse genes (10,724), and 8,358 of those were determined to be full-length reconstructable [5]. In our study, Trinity was applied to assemble rhesus transcripts with a RNA-Seq data for a brief comparison with our proposed method which uses different data sources.

### *Ab initio* and evidence-driven gene prediction

*ab initio* gene predictors use mathematical models to identify genes and their structures [10], without external evidences. However, training is very important to *ab initio* gene predictors, which requires high-quality gene models or deep understanding with organism-specific genomic traits (such as codon frequencies, GC contents and distributions of intron-exon lengths). Therefore, most *ab initio* gene predictors require precalculated parameter files for model organisms. Therefore, the external evidence, such as alignments of ESTs, RNA-Seq data, and protein sequences are used to train gene predictors or to improve the accuracy of predictions. That is the evidence-driven gene prediction. MAKER pipeline [9] uses EST, protein and mRNA-Seq alignments to train the predictors Augustus [11,12] and SNAP [13]. Augustus, SNAP and Gnomon [14] also use external evidence to improve the quality of gene prediction, such as ESTs for exon boundaries.

The above methods still require collecting or pre-creating different data sources, such as protein sequences, ESTs, and RNA-Seq datasets for alignments or model trainings to synthesize the final genome structure annotations. In this study, our goal is to offer, with good accuracy and sufficient abundance, a quick view of genome structural annotations for an un-annotated eukaryotic genome based on an annotated similar species. Our method, named GASS (Genome Annotation based on the Species Similarity), quickly annotated rhesus genome with human genome’ annotations (5 days for Basic Local Alignment Search Tool(BLAST) running [15] and 1.5 hours for GASS running). The annotation process does not require extra protein sequences, ESTs, or RNA-Seq datasets, which greatly reduces time and money costs. Firstly, Exon sequences in the annotated species are imported into BLAST to find the similar segments in the unannotated genome. One exon sequence is possible to be aligned to multiple regions in the unannotated genome. Hence, for a transcript containing several exons, there are a large number of combinations of aligned-segments. And then, GASS is used to find the optimal combination of the aligned-segments in the unannotated genome to integrate one transcript’s annotation. After quality control, GASS builds structural annotations for the unannotated species. In our study, GASS was used to annotate the rhesus genome structure based on the 97.5% identity at nucleotide and amino acid sequence levels between rhesus and human [16]. The rhesus annotations produced by GASS was evaluated by comparing it to the two existing rhesus annotation databases, RefSeq [17] and Ensembl [18], and being aligned with RNA-Seq alignments. GASS found more than 65% RefSeq-rheMac3’s exons exactly and has better specificities than Ensembl-rheMac2 at genes, transcripts and exons levels for almost all testing RNA-Seq datasets. Furthermore, we discovered numerous 2 bp annotating shifts on one side of exon boundaries between RefSeq and GASS annotations, which were concomitant with the incomplete “GT-AG” canonical splicing sites in RefSeq. Detailed analysis showed that the 2 bp shifts and incomplete splicing sites were led by the mis-assemblies of the rheMac3 genome. The conclusion was further supported by the alignments of three RNA-Seq and two DNA-Seq datasets; and the corresponding amino acids sequences in RefSeq. Our experiments demonstrate that GASS provides an easy-to-use, efficient, time and cost saving method to annotate a eukaryotic genome sequence.

## Methods

**Figure 1:**
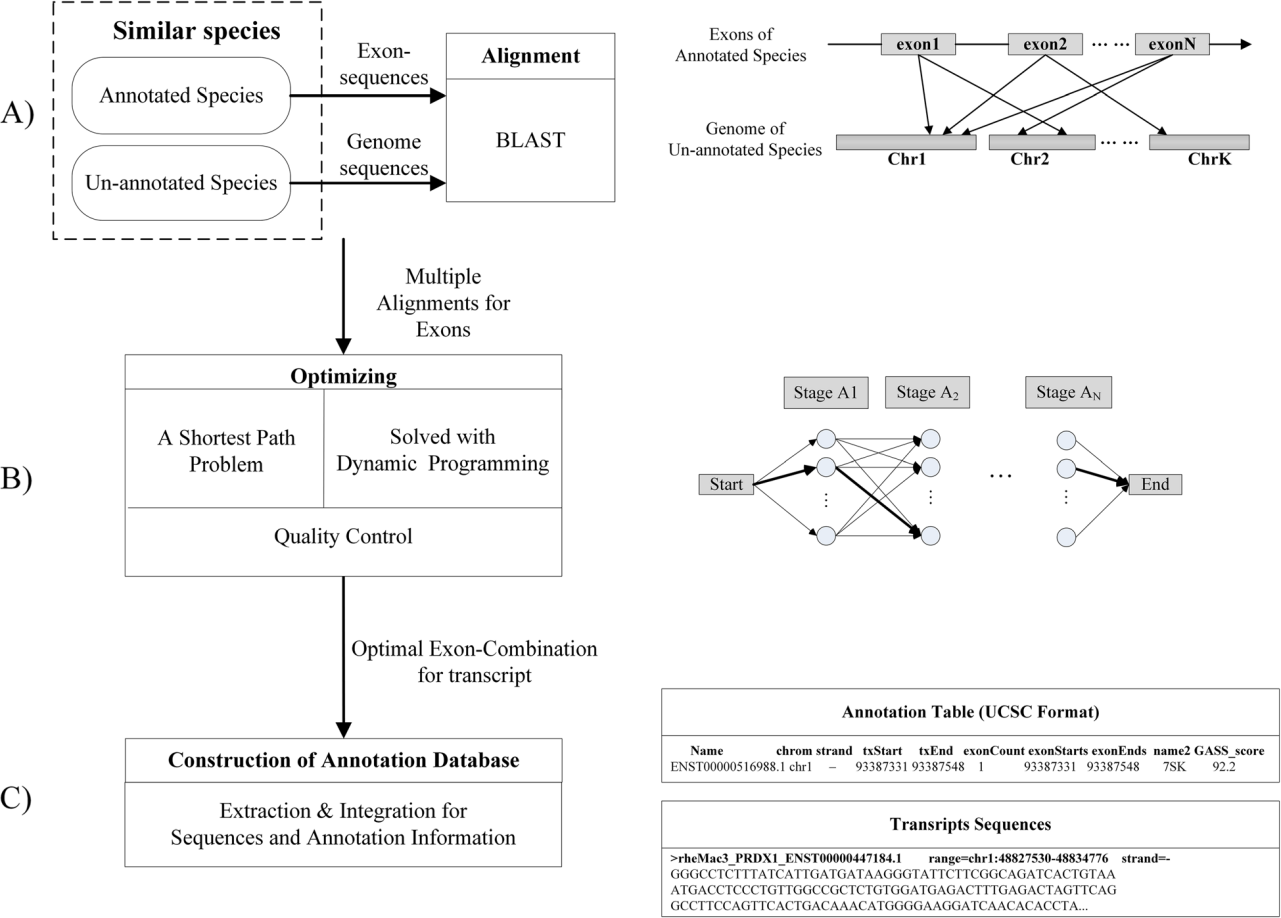
**GASS processing pipeline. A)** exon-sequences of a transcript from *AG* are aligned to the un-annotated genome with BLAST. And each exon might be aligned to multiple similar regions in *UG*. **B)** the search for optimal exon-combination for a transcript is modelled as a shortest path problem. This problem can be solved with dynamic programming algorithm. **C)** after quality control, the transcript annotations and the corresponding sequences are extracted from the un-annotated genome sequences and organized as UCSC genome format.

GASS produces the genome annotations with the processing pipeline shown in Figure 1. ① Exon-sequences (denoted as *E*_*T*_) of a transcript (denoted as *T*) of the annotated species (denoted as *AG*) are aligned to the un-annotated genome (denoted as *UG*) with BLAST, as in Figure 1(A). The purpose is to find the similar regions in *UG* to *E*_*T*_ in *AG*. And each exon might have multiple similar regions in *UG*. ② As in Figure 1(B), the search for optimal exon-combination for a transcript is modelled as a shortest path problem. This problem can be solved with dynamic programming algorithm. ③ As in Figure 1(C), after quality control, the transcript annotations and the corresponding sequences are extracted from the un-annotated genome sequences and organized as UCSC genome format [19]. The detail of each step is described in the following subsections.

### Pre-processing: alignments from the annotated exons to the un-annotated genome with BLAST

The exon-sequences of *AG* are aligned to *UG* with BLAST. For each exon, BLAST outputs statistically significant similar segments in the *UG*. The alignments find highly similar regions between *AG* and *UG*. The BLAST running configurations are listed in Section 1 of the Supplementary.

Eukaryotic genomes can be highly repeat rich [1]; therefore, one exon-sequence might be aligned to large numbers of regions in *UG*. For example, one human exon-sequence might generally be found similar to more than 100, and even to 50,000 regions of rhesus genome. Thus, for a transcript with 10 exons, there might be more than 10^10^ possible aligned-exon combinations. An optimization model and evaluation metrics are required to select a proper combination to integrate a transcript annotation.

### Problem description: A shortest path model

For *AG*, if all the exons of a transcript *E*_*T*_ are aligned to *UG* with sufficient accuracy and proper position relationship, there is high confidence to tell a similar transcript existing in *UG*.

However, just as we mentioned above, there are large numbers of possible aligned-exon combinations for a transcript in *UG*. Then selecting an optimal transcript annotation requires proper evaluating measurements and selection model.

**Figure 2:**
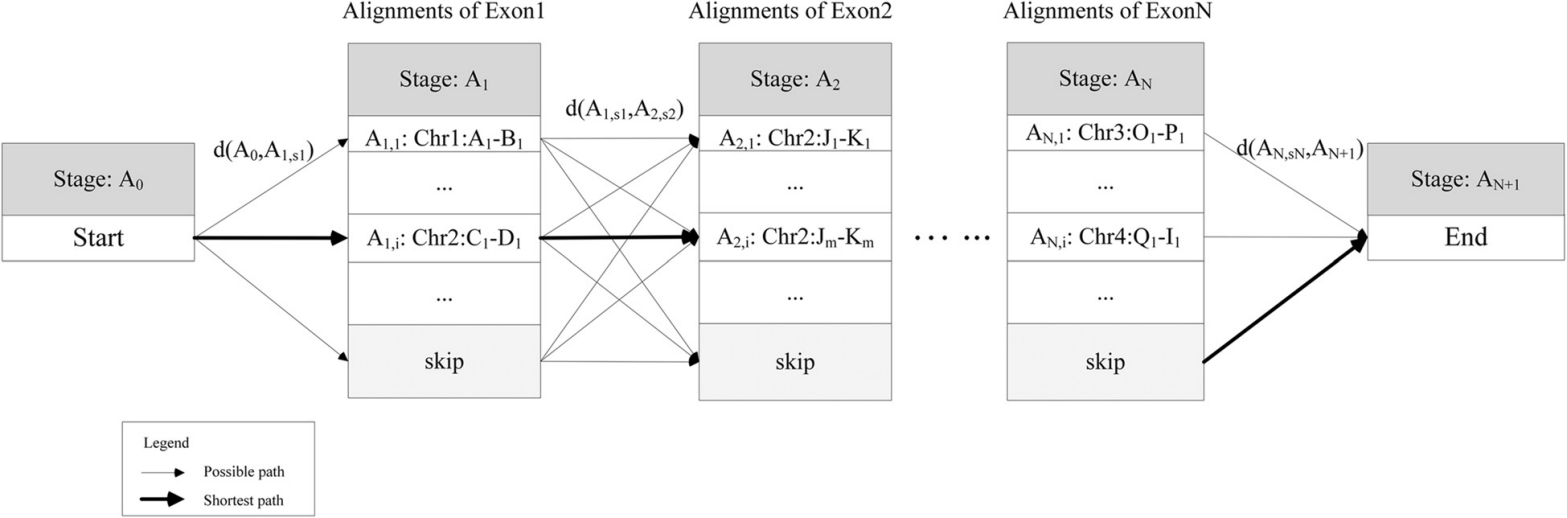
**The shortest path model for the optimization of alignment-combinations.** *Exon i* (*i = 1…N*) in *AG* are aligned to maybe multiple similar regions in *UG*. For example, *Exon 1* is mapped to *Chr1:A*_*1*_ *-B*_*1*_; … ;*Chr2:C*_*1*_ *-D*_*1*_; …, where *Chr1:A*_*1*_ *-B*_*1*_ means *Exon 1* is aligned from *A*_*1*_ to *B*_*1*_ in *Chromosome 1* of *UG*. All alignments of *Exon i* is denoted as *stage A*_*i*_, and the *j* ^*th*^ alignment of *Exon i* is denoted as *A*_*i,j*_. *d*(*A*_*i*,*k*_, *A*_*i* + 1,*p*_) measures the quality of Alignment *A*_*i,k*_ and the rationality of relationship between *A*_*i,k*_ and *A*_*i+1,p*_ for neighbour *Exon i* and *Exon i + 1*. The stages “*start*” and “*end*” are the beginning and termination of one exon-combination path. The state “*skip*” is an extra state for each exon-alignment stage for the cases that there is no reasonable alignment available for one certain exon.

The multiple exon-alignments and exon-combinations are described as Figure 2.

① *Exon i* (*i = 1…N*) in *AG* are aligned to *UG* and each exon might have multiple similar regions in *UG*. For example, *Exon 1* is mapped to *Chr1:A*_*1*_*-B*_*1*_; … ;*Chr2:C*_*1*_*-D*_*1*_; …, where *Chr1:A*_*1*_*-B*_*1*_ means *Exon 1* is aligned from *A*_*1*_ to *B*_*1*_ in *Chromosome 1* of *UG*.

② The stages “*start*” and “*end*” indicate the starting and ending of one combination of exon-alignments along with the chromosome direction. The combination is a candidate annotation of a transcript. When the strand direction of source-transcript in *AG* is “-”, the stages “*start*” and “*end*” reversely correspond to the transcript termination and starting sites respectively.

③ When the corresponding relationship between chromosomes from *AG* and *UG* is available, the stage “*start*” is set as a fixed chromosome, which limits the follow-up alignments coming from this pre-set chromosome. Otherwise, there is no limitation for the stage “*start*”.

④ All alignments of *Exon i* is denoted as *stage A*_*i*_. The alignment being studied is called “*state*” (denoted as *s*_*i*_), and *s*_*i*_ = *j* means the *j*^*th*^ alignment. ${A}_{i,{s}_i}$ means the *s*_*i*_^*th*^ alignment of *Exon i*. The state “*skip*” is an extra state for each exon alignment stage for the cases that there is no reasonable alignment available for one certain exon.

⑤ $d\left({A}_{i,{s}_i},{A}_{i+1,{s}_{i+1}}\right)$ measures the quality of Alignment ${A}_{i,{s}_i}$ and its relationship with the next stage ${A}_{i+1,{s}_{i+1}}$, that is the rationality for alignment ${A}_{i,{s}_i}$ and ${A}_{i+1,{s}_{i+1}}$ being two neighbour Exons.

⑥ The smaller $d\left({A}_{i,{s}_i},{A}_{i+1,{s}_{i+1}}\right)$ is, the bigger possibility that the ${A}_{i,{s}_i}$ and ${A}_{i+1,{s}_{i+1}}$ compose two neighbour exons in the transcript is. $d\left({A}_{i,{s}_i},{A}_{i+1,{s}_{i+1}}\right)=0$ means perfect ${A}_{i,{s}_i}$ alignments and the most rational neighbour exons relationship.

Therefore, the optimal exon-combination for one transcript in *UG* is represented as:

1 $$ \begin{array}{l}\underset{s_i\ i=1\cdots N}{ \min}\left\{d\left({A}_{1,{s}_1},{A}_{2,{s}_2}\right)+d\left({A}_{2,{s}_2},{A}_{3,{s}_3}\right)+\cdots +d\left({A}_{N-1,{s}_{N-1}},{A}_{N,{s}_N}\right)\right\}\\ {}=\underset{s_i\ i=1\cdots N}{ \min }{\displaystyle \sum_{i=1}^{N-1}d\left({A}_{i,{s}_i},{A}_{i+1,{s}_{i+1}}\right)}\kern0.5em \end{array} $$

where *s*_*i*_ = 1…*i*_*M*_ , *i*_*M*_ is the number of similar regions (alignments) in *UG* for *Exon i*.

This is a typical shortest path problem, whose goal is to plan a shortest path with a fix starting point, passing through *N* decision-stages and reaching a fixed termination point. There are large numbers of candidate paths with various $d\left({A}_{i,{s}_i},{A}_{i+1,{s}_{i+1}}\right)$ from ${A}_{i,{s}_i}$ to the next stage ${A}_{i+1,{s}_{i+1}}$. $d\left({A}_{i,{s}_i},{A}_{i+1,{s}_{i+1}}\right)$ is called “*one-step cost*” in the shortest path model and its detail definition is given in the subsection “Definition of *one-step cost* of the shortest path model”.

In our study, the shortest path problem is explained as: searching for an optimal exon-combination is to find a path, which links certain exon-alignment at each stage to obtain the overall optimal alignments and rational relationships, that is, with minimum sum of *one-step costs*.

The shortest path problem can be solved with the classical dynamic programming algorithm, which is described briefly in the next subsection.

### Problem solution: dynamic programming algorithm

For a shortest path problem, decision at each stage results in immediate *one-step cost* but also affects the context in which future decisions are to be made and therefore affects the cost incurred in future stages. The optimization of the shortest path model is to minimize the total cost over all the decision stages. It is challenging because of the trade-off between immediate and future costs. Dynamic programming algorithm breaks the optimization problem into simpler sub-problems. The original optimal problem is turned in the following Bellman’s equation with recursive relationship, as in Formula (2).

2 $$ \left\{\begin{array}{c}\hfill \kern0.95em {f}^{*}\left({A}_{i,{s}_i}\right)=\underset{s_{i+1}}{ \min}\left({f}^{*}\left({A}_{i+1,{s}_{i+1}}\right)+d\left({A}_{i,{s}_i},{A}_{i+1,{s}_{i+1}}\right)\right)\kern0.10em ,{s}_{i+1}=1\dots {\left(i+1\right)}_M,\ i=1\dots N\hfill \\ {}\hfill {f}^{*}\left({A}_{N+1}\right)=0,\ {A}_{N+1}\ \mathrm{is}\  End\ \mathrm{point}\hfill \\ {}\hfill {f}^{*}\left({A}_0\right)=\underset{s_1}{ \min}\left({f}^{*}\left({A}_{1,{s}_1}\right)\right), {s}_1=1..{.1}_M,{A}_0\mathrm{is}\  Start\ \mathrm{point}\hfill \end{array}\right. $$

where ① ${f}^{*}\left({A}_{i,{s}_i}\right)$ is the shortest path starting from ${A}_{i,{s}_i}$ (the *s*_*i*_^*th*^ alignment of *Exon i*) to the ending point. ② The basic idea to solve the shortest path problem with dynamic programming is: within a complete shortest path, the sub-path from node *j* to *q* is the shortest path from *j* to *q*. ③ f*(*A*_*N* + 1_) = 0 is the boundary cost. ④ Formula (2) is the recursive solution implemented in reverse for the shortest path problem. ⑤ The shortest length from *A*_*1,k*_ to the end point is ${f}^{*}\left({A}_{1,{s}_1}\right)$. The overall optimal cost f*(*A*_0_) is the minimum value among all shortest paths starting from stage *A*_*1*_.

The optimal shortest path is normalized with Formula (3). The optimal *Score* for one transcript is 100. The optimal path selects proper aligned segments to build a transcript annotation.

3 $$ Score=\left(1-\frac{f^{*}\left({A}_0\right)}{N}\right)\times 100\% $$

### Definition of *one-step cost*$d\left({A}_{i,{s}_i},{A}_{i+1,{s}_{i+1}}\right)$ of the shortest path model

$d\left({A}_{i,{s}_i},{A}_{i+1,{s}_{i+1}}\right)$ is the immediate *one-step cost* when the path from alignment *s*_*i*_ of stage *i* to the alignment *s*_*i+1*_ of stage *i + 1* is selected. In our study, $d\left({A}_{i,{s}_i},{A}_{i+1,{s}_{i+1}}\right)$ should measure the identity of alignment *A*_*i,k*_ and the rationality that regions *A*_*i,k*_ and *A*_*i+1,p*_ compose two neighbour exons in one transcript.

In our study, the following factors are taken into consideration for $d\left({A}_{i,{s}_i},{A}_{i+1,{s}_{i+1}}\right)$:

① Each exon *E*_*T*_ from transcript *T* of *AG* should be aligned to one common chromosome of *UG*. (*Chr_dist*: Chromosome restriction)

② Alignments of all exons of one source transcript *T*, that is ${A}_{i,{s}_i}$, *s*_*i*_ = 1 ⋯ *i*_*M*_, should keep same strand directions in *UG*. (*Strand*: Strand direction restriction)

③ The exons from *AG* should be aligned to *UG* with sufficient accuracy and confidence. (*Align_Rela*: Alignment evaluation)

④ The position distance of two neighbour exons in *UG* should be comparable to that in *AG*. (*Align_Rela*: Relationship restriction of two aligned neighbour regions)

⑤ The exons’ order in *UG* should remain same with their order in the AG, and this restriction is ensured by the construction of the shortest path model.

Considering the above five factors, one-step cost *d*(*A*_*i*,*k*_, *A*_*i* + 1,*p*_) is defined as formula (4) for “*alignment*” and “*skip*” states respectively:

4 $$ d\left({A}_{i,{s}_i},{A}_{i+1,{s}_{i+1}}\right)=\begin{array}{cc}\hfill \left\{\begin{array}{l}Chr\_ dist\left({A}_{i,{s}_i},{A}_{i+1,{s}_{i+1}}\right)+ Strand\left({A}_{i,{s}_i},{A}_{i+1,{s}_{i+1}}\right)+ Align\_ Rela\left({A}_{i,{s}_i},{A}_{i+1,{s}_{i+1}}\right),\\ {}K,\end{array}\right.\hfill & \hfill \begin{array}{l}{A}_{i,{s}_i}\ \mathrm{is}\  alignment\\ {}{A}_{i,{s}_i}\ \mathrm{is}\  skip\end{array}\hfill \end{array} $$

① $Chr\_ dist\left({A}_{i,{s}_i},{A}_{i+1,{s}_{i+1}}\right)$ measures whether the current two neighbour alignments of *Exon_i-*1 and *Exon_i* are from a common chromosome, as shown in Formula (5). If the two alignments are from the same chromosome, $Chr\_ dist\left({A}_{i,{s}_i},{A}_{i+1,{s}_{i+1}}\right)=0$; otherwise, the $Chr\_ dist\left({A}_{i,{s}_i},{A}_{i+1,{s}_{i+1}}\right)=\infty$.

5 $$ Chr\_ dist\left({A}_{i,{s}_i},{A}_{i+1,{s}_{i+1}}\right)=\left\{\begin{array}{l}0, \kern0.5em {A}_{i,{s}_i}\ \mathrm{a}\mathrm{nd}\ {A}_{i+1,{s}_{i+1}}\ \mathrm{a}\mathrm{re}\ \mathrm{mapped}\ \mathrm{t}\mathrm{o}\ \mathrm{a}\ \mathrm{common}\ \mathrm{chromosome}\\ {}\infty, \kern0.5em {A}_{i,{s}_i}\ \mathrm{a}\mathrm{nd}\ {A}_{i+1,{s}_{i+1}}\ \mathrm{a}\mathrm{re}\ \mathrm{mapped}\ \mathrm{t}\mathrm{o}\ \mathrm{different}\ \mathrm{chromosome}\mathrm{s}\end{array}\right. $$

② $Strand\left({A}_{i,{s}_i},{A}_{i+1,{s}_{i+1}}\right)$ ensures the current two neighbour alignments of *Exon_i-*1 and *Exon_i* are in the same direction, as shown in Formula (6). If the two alignments are in the same direction, $Strand\left({A}_{i,{s}_i},{A}_{i+1,{s}_{i+1}}\right)=0$; otherwise, $Strand\left({A}_{i,{s}_i},{A}_{i+1,{s}_{i+1}}\right)=\infty$.

6 $$ Strand\left({A}_{i,{s}_i},{A}_{i+1,{s}_{i+1}}\right)=\left\{\begin{array}{l}0, \kern0.5em {A}_{i,{s}_i}\ \mathrm{and}\ {A}_{i+1,{s}_{i+1}}\ \mathrm{are}\ \mathrm{in}\ \mathrm{the}\ \mathrm{s}\mathrm{ame}\ \mathrm{direction}\\ {}\infty, \kern0.5em {A}_{i,{s}_i}\ \mathrm{and}\ {A}_{i+1,{s}_{i+1}}\ \mathrm{are}\ \mathrm{in}\ \mathrm{the}\ \mathrm{different}\ \mathrm{direction}\mathrm{s}\end{array}\right. $$

③ $Align\_ Rela\left({A}_{i,{s}_i},{A}_{i+1,{s}_{i+1}}\right)$ measures the alignment quality and the position relationship between the alignments of two neighbour exons, as defined in Formula (7).

7 $$ Align\_ Rela\left({A}_{i,{s}_i},{A}_{i+1,{s}_{i+1}}\right)=1- Align\left({A}_{i,{s}_i}\right)* Rela\left({A}_{i,{s}_i},{A}_{i+1,{s}_{i+1}}\right) $$

8 $$ Align\left({A}_{i,{s}_i}\right)=\frac{Align\_ Length\left({A}_{i,{s}_i}\right)}{Exon\_ length\left({A}_{i,{s}_i}\right)}{e}^{-E\left({A}_{i,{s}_i}\right)} $$

9 $$ Rela\left({A}_{i,{s}_i},{A}_{i+1,{s}_{i+1}}\right)=\frac{ \min \left(\left| Distance\left(Exo{n}_i,Exo{n}_{i+1}\right)\right|,\left| Distance\left({A}_{i,{s}_i},{A}_{i+1,{s}_{i+1}}\right)\right|\right)}{ \max \left(\left| Distance\left(Exo{n}_i,Exo{n}_{i+1}\right)\right|,\left| Distance\left({A}_{i,{s}_i},{A}_{i+1,{s}_{i+1}}\right)\right|\right)} $$

$Align\left({A}_{i,{s}_i}\right)$ measures the quality of alignment ${A}_{i,{s}_i}$ to the *UG*, as in Formula (8). $Align\_ Length\left({A}_{i,{s}_i}\right)$ is the number of nucleotides in alignment ${A}_{i,{s}_i}$ from *Exon i* to *UG*. $Exon\_ Length\left({A}_{i,{s}_i}\right)$ is the full length of *Exon i* in *AG*. $E\left({A}_{i,{s}_i}\right)$ is the *Evalue* of the alignment of *Exon i* from BLAST and ${e}^{-E\left({A}_{i,{s}_i}\right)}$ measures the confidence and the significance of the alignment ${A}_{i,{s}_i}$. The value of $Align\left({A}_{i,{s}_i}\right)$ is in [0,1]. $Align\left({A}_{i,{s}_i}\right)=1$ means perfect alignments from *Exon i* to *UG*.

$Rela\left({A}_{i,{s}_i},{A}_{i+1,{s}_{i+1}}\right)$ measures distances ratio of the two aligned segments in *UG* and the two source exons in *AG*, as shown in Formula (9). |*Distance*(*Exon*_*i*_, *Exon*_*i* + 1_)| is the intron length between *Exon i* and *Exon i + 1*. $\left| Distance\left({A}_{i,{s}_i},{A}_{i+1,{s}_{i+1}}\right)\right|$ is the nucleotide length between the ending of alignment ${A}_{i,{s}_i}$ and the starting of alignment ${A}_{i+1,{s}_{i+1}}$. If the two distances are very close, the two aligned segments are very possible to be neighbour exons. The value of $Rela\left({A}_{i,{s}_i},{A}_{i+1,{s}_{i+1}}\right)$ is in [0,1]. The best value of $Rela\left({A}_{i,{s}_i},{A}_{i+1,{s}_{i+1}}\right)$ is 1.

$Align\left({A}_{i,{s}_i}\right)* Rela\left({A}_{i,{s}_i},{A}_{i+1,{s}_{i+1}}\right)$ requires both $Align\left({A}_{i,{s}_i}\right)$ and $Rela\left({A}_{i,{s}_i},{A}_{i+1,{s}_{i+1}}\right)$ are very close to 1, that is, good alignment and position relationship. If $Align\left({A}_{i,{s}_i}\right)=0.2$ and $Rela\left({A}_{i,{s}_i},{A}_{i+1,{s}_{i+1}}\right)=1$, from intuition, it is not a reasonable decision. $Align\left({A}_{i,{s}_i}\right)* Rela\left({A}_{i,{s}_i},{A}_{i+1,{s}_{i+1}}\right)=0.2$ is a poor evaluation score. However, if we use $\frac{Align\left({A}_{i,{s}_i}\right)+ Rela\left({A}_{i,{s}_i},{A}_{i+1,{s}_{i+1}}\right)}{2}=0.6$, the score infers that it is still an acceptable decision. Therefore, multiplying the two items means more stringent requirement for the evaluations.

Therefore, $Align\_ Rela\left({A}_{i,{s}_i},{A}_{i+1,{s}_{i+1}}\right)\in \left[0,1\right]$ and $Align\_ Rela\left({A}_{i,{s}_i},{A}_{i+1,{s}_{i+1}}\right)=0$ means best alignments and position relationship. The minimum *one-step cost*$d\left({A}_{i,{s}_i},{A}_{i+1,{s}_{i+1}}\right)=0$.

Especially, when for all alignments *s*_*i*_ in *Exon i* and *s*_*i+1*_ in *Exon i + 1*, $Chr\_ dist\left({A}_{i,{s}_i},{A}_{i+1,{s}_{i+1}}\right)=\infty$, $Strand\left({A}_{i,{s}_i},{A}_{i+1,{s}_{i+1}}\right)=\infty$ or $Align\_ Rela\left({A}_{i,{s}_i},{A}_{i+1,{s}_{i+1}}\right)$ is close to 1, there is no possibility to link two alignments as neighbour exons. The state “*skip*” is selected for *Exon i* and the one-step cost $d\left({A}_{i,{s}_i},{A}_{i+1,{s}_{i+1}}\right)=K$. The constant *K* is set at 0.95, which is the punishment for skipping stage *i*. The state “*skip*” keeps the chromosome information of the previous stage.

### Quality control for annotations

For the selected optimal alignment-combinations, the following criteria were used to keep high-quality annotations:

① Checking for canonical GT-AG donor and acceptor splicing sites. For eukaryotes, 98.71% of splicing junctions contain the canonical “GT-AG” splicing sites [20]. Therefore, GASS checks whether the splice sites contain the GT and AG dinucleotide motif, as shown in Additional file 1: Figure S2. If GASS cannot find the GT-AG donor and acceptor in the introns’ boundary of one optimal alignment-combination, 10 bp two-side shifts are extended to search the canonical splice site. After that, if GASS still cannot find the canonical splicing site, the annotation are marked extra “N” in the last column of the annotation table.

② Filtering out the low-score exon-combinations. GASS only keeps the annotations whose scores of the shortest paths are more than 80. It is a clear indication of high quality of the annotation.

GASS provides the annotated transcript sequences in FASTA format, and the annotations information with the table format of UCSC Genome Bioinformatics [19].

### Software implementation

GASS is coded with Python and implemented as a processing pipeline with UNIX shell. The GASS Pseudo code is shown in Additional file 1: Figure S3. The GASS source codes are available at http://gassflow.codeplex.com/.

## Results and discussions

To evaluate its performance, GASS was applied to annotate the genome of Macaca mulatta (rhesus) based on 97.5% identity at nucleotide and amino acid sequence levels between rhesus and Homo Sapiens (human) [16]. We selected rhesus genome for annotation due to the following reasons:

① There are several public rhesus annotation databases, which are available to evaluate GASS annotations as reference information.

② According to the existing study, the current rhesus annotation is far from satisfactory [21-23] and at least 28.7% of the rhesus transcripts were mis-annotated [23]. If GASS could produce more accurate annotations on the well-annotated human genome, it would be helpful for related studies.

### Experiment design

The rhesus genome was assembled as rheMac2 (published on Jan. 2006) and rheMac3 (published on Oct. 2010). The human genome was well-assembled and well-annotated, such as GENCODE [24] Genes track on GRch37/hg19. Therefore, in our study, rheMac3 genome was annotated on the human GENCODE V14 annotations. The exons’ sequences from GENCODE were aligned to rheMac3 genome with BLAST. The rhesus annotations were produced by GASS. Two existing rhesus annotation databases were compared to GASS annotations: RefSeq for rheMac3 genome built by NCBI (called “RefSeq-rheMac3”); Ensembl for rheMac2 genome built by Ensembl (called “Ensembl-rheMac2”). RefSeq is well known for its most conservative and precise annotations. Ensembl produces the annotations automatically and manually.

BLAST running parameters in our study are described in Section 1 of Supplementary. It took five days to produce the alignment results with BLAST. The result for each human chromosome was approximately 30GB in file size. It took GASS approximately 1.5 hours (with parallel running) to process the alignment files to achieve the structural annotations for the rhesus genome. The produced rhesus annotations are available at http://gassflow.codeplex.com/.

### Summary of the rhesus annotations produced by GASS

After quality control, GASS produced 22,416 genes, 60,730 transcripts, 210,495 exons, 158,756 splicing junctions and 95,339,302 non-overlap nucleotide bases. The amount of produced elements from GASS, RefSeq-rheMac3 and Ensembl-rheMac2 are given in Table 1. The annotated elements produced by GASS are much more than that from RefSeq-rheMac3 (22,416 vs. 6,274). Ensembl-rheMac2 annotated about 6,000 more genes than GASS, but the transcripts are less than that in GASS.

**Table 1:** **Summary of the three rhesus annotation**

**Items**	**Genes**	**Transcripts**	**Isoforms** ^**1**^	**Exons** ^**2**^	**Splicing–junctions** ^**2**^	**Nucleotide bases** ^**3**^
GASS	22,416	60,730	48,882	210,495	158,756	95,339,302
RefSeq-rheMac3	6,274	6,360	156	45,540	39,230	114,849,69
Ensembl-rheMac2	28,595	42,820	22,156	239,754	200,748	70,038,189

^1^Isoforms: if a gene has more than one transcript, we called these transcripts isoforms.

^2^If several exons/splicing junctions share identical splice donor and acceptor sites, we count the exons/splicing junctions only once.

^3^The nucleotide bases are the union of the isoforms.

### Direct comparison to RefSeq-rheMac3

Based on rheMac3 genome, annotations from RefSeq-rheMac3 and GASS were compared directly. The Ensembl-rheMac2 was excluded because it was annotated on rheMac2 genome. Two generations of genome assemblies, rheMac2 and rheMac3, lead to different coordinate system, which cannot be compared directly. Rhesus and human share the same gene-naming system, so 3,647 common genes from RefSeq and GASS were extracted as a baseline for further comparisons at exon, splicing junction, and transcript levels. The amounts of these elements on the common genes are shown in Additional file 1: Table S1.

**Figure 3:**
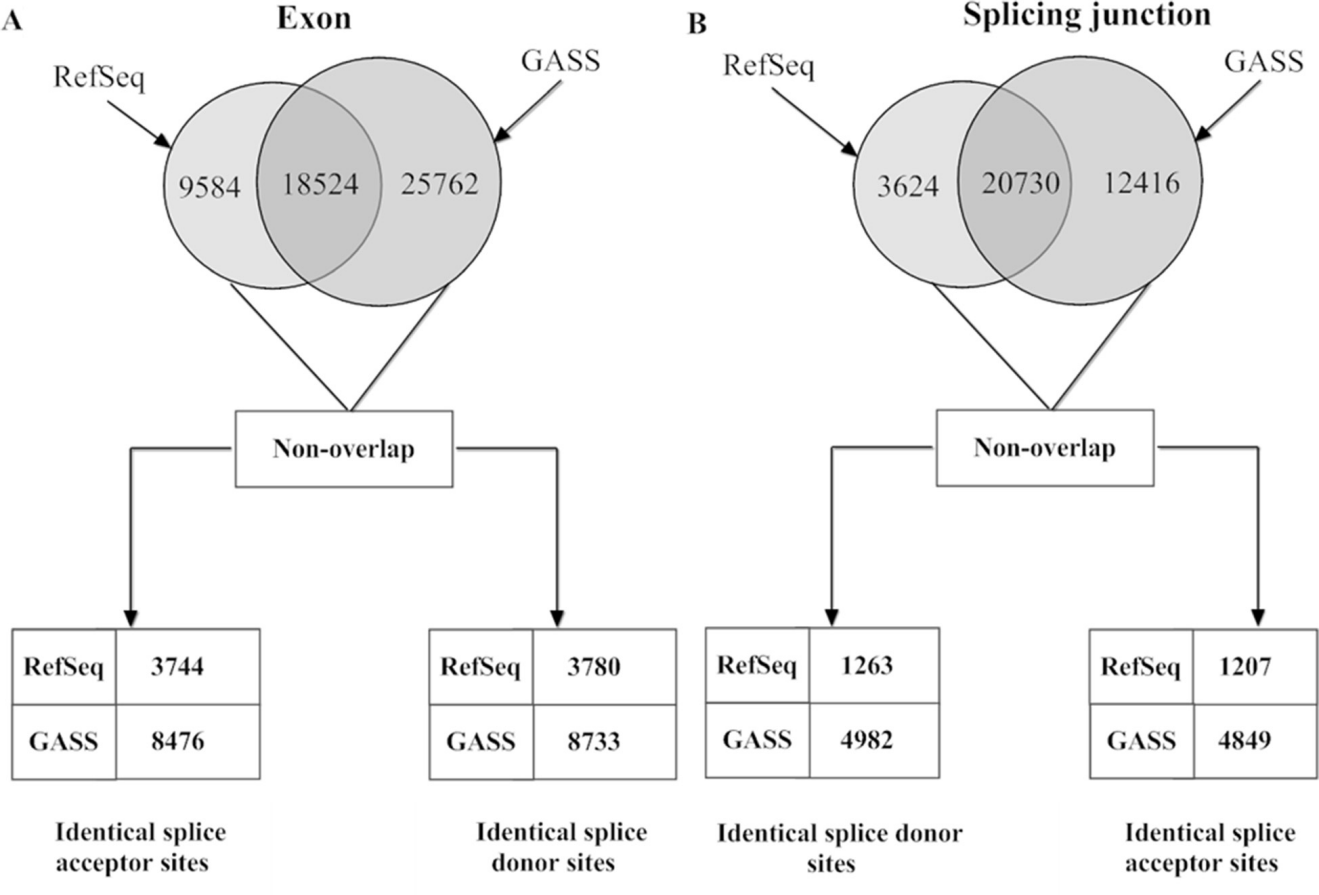
**Comparing GASS and RefSeq-rheMac3 at exon and splicing junction level. A)** The comparison of exons from GASS and RefSeq-rheMac3. The overlap of the two sets is the amount of common exons sharing by the two databases. Among the non-overlap exons, the amounts of exons sharing identical splice acceptors or donors are given. **B)** The comparison of splicing junctions from GASS and RefSeq-rheMac3. The overlap of the two sets is the amount of common splicing junctions sharing by the two databases. Among the non-overlap splicing junctions, the amounts of splicing junctions sharing identical donors or acceptors are given.

**Exon level:** If two exons share identical boundaries, the exons are considered as same exons. As shown in Figure 3(A), 65.9% of RefSeq-rheMac3’s exons are exactly same with GASS’s exons. Moreover, 26.8% of RefSeq-rheMac3’s exons share common splice acceptors or donors with GASS’s exons.

**Splicing junction level:** As shown in Figure 3(B), 85.12% of RefSeq-rheMac3’s splicing junctions are exactly same with GASS’s splicing junctions. 10.1% of RefSeq-rheMac3’s splicing junctions share common splice donors or acceptors with GASS’s splicing junctions.

**Figure 4:**
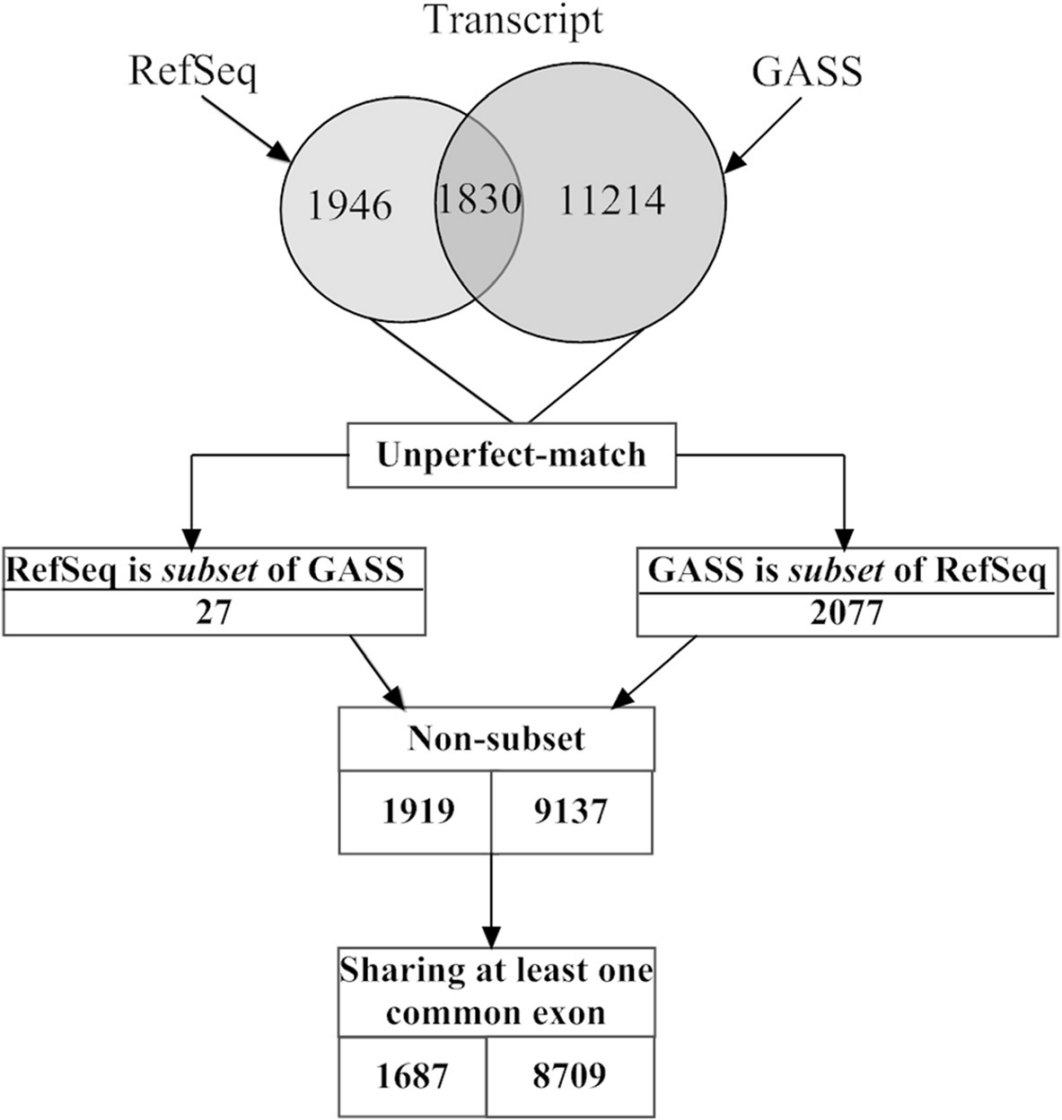
**Comparing GASS and RefSeq-rheMac3 at transcript level.** The overlap of the two sets is the amount of exactly same transcripts. Among the non-identical transcripts, there are 2,077 GASS transcripts, and each one is the “*subset*” of RefSeq-rheMac3’s certain transcript. And there are 27 RefSeq-rheMac3 transcripts, and each one is the “*subset*” of GASS’s certain transcript. The amount of transcripts sharing at least one identical exon is also analysed.

**Transcript level:** The UTR 5’ and 3’ regions are the most imprecise parts of gene annotations [25]. Therefore, we excluded the first and the last exons from each transcript during the comparison. As shown in Figure 4, approximately 50% of RefSeq-rheMac3’s transcripts are exactly same with GASS’s transcripts. For transcript *A*, if all the exons can be found in transcript *B*, but transcript *A* misses exon(s) in transcript *B*, then transcript *A* is a “*subset*” of transcript *B.* There are 2,077 GASS transcripts and each one is the “*subset*” of RefSeq-rheMac3’s transcript. And there are 27 RefSeq-rheMac3 transcripts and each one is the “*subset*” of GASS’s transcript, as illustrated by Additional file 1: Figure S4. And 1,687 Refseq transcripts and 8,709 GASS transcripts share at least one common exon. That is, 87.91% of RefSeq-rheMac3’s transcripts share at least one exon with transcripts in GASS.

To give a baseline that how similar two public annotation databases for same species would be, Refseq-rheMac2 and Ensembl-rheMac2 databases were also compared directly. The results are given in Additional file 1: Table S2, Figure S5, Figure S6 and Figure S7. The two databases share 2,631 common genes. At exon level, 72.2% of RefSeq-rheMac2’s exons are exactly same with Ensembl-rheMas2’s exon, and 21.1% of RefSeq-rheMac2’s exons share common splice acceptors and donors with Ensembl-rheMac2’s exons. At junction level, 91.9% of RefSeq-rheMac2 splicing junctions are exactly same with Ensembl-rheMac2’s splicing junctions, and 2.02% of RefSeq-rheMac2’s splicing junctions share common donors or acceptors points with Ensembl-rheMac2’s splicing junctions. At transcript level, about 50% of the RefSeq-rheMac2 transcripts are exactly same with Ensembl-rheMac2’s transcripts and 66.56% of RefSeq-rheMac2’s transcripts share at least one exon with Ensembl-rheMac2’s transcripts.

From the above results, we can see that the similarity between GASS and RefSeq-rheMac3 are comparable to that between Refseq-rheMac2 and Ensembl-rheMac2, which means the annotations produced by GASS have comparable accuracy with the public annotation database.

**Figure 5:**
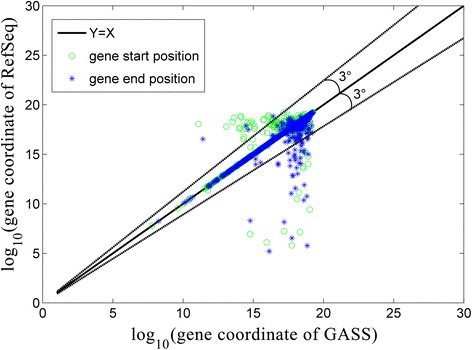
**Genes’ boundaries in RefSeq-rheMac3 and GASS.** The genes’ boundaries of GASS and RefSeq-rheMac3 are designated in X-axis and Y-axis for comparison respectively. Almost all the dots are highly close to the line Y = X, which means that the genes’ boundaries from GASS and RefSeq-rheMac3 are highly identical. The logarithm is applied to re-scale the coordinates. The black sold line is an offset of 3° and 95% of the points are close to the sold line.

We also compared the transcripts’ starting and termination sites identified in RefSeq-rheMac3 and GASS for the 3,647 common genes. The transcripts starting and termination positions are marked with circles and stars in Figure 5, respectively. The transcripts’ boundaries of GASS and RefSeq-rheMac3 are designated in X-axis and Y-axis respectively. Almost all the dots are highly close to the line Y = X, which means that the transcripts’ starting and termination sites from GASS and RefSeq-rheMac3 are highly identical.

### Evaluation with RNA-Seq datasets

RNA-Seq data contains accurate and abundant information to support the splicing junctions, exons and transcripts. And GAAP [26], a software pipeline, was previously developed to evaluate the accuracy and completeness of genome annotation databases with RNA-Seq datasets. Therefore, the rhesus annotations produced by GASS was evaluated with rhesus RNA-Seq datasets by GAAP pipeline in this section.

There are 10,096 common genes between Ensembl-rheMac2 and GASS. These genes were extracted to offer a baseline for comparison. The amounts of exons, splicing junctions, transcripts of the common genes are shown in Additional file 1: Table S3. However, for Refseq-rheMac3, we used all the genes because of its conservation in annotations. Three rhesus RNA-Seq datasets in Table 2 were aligned to the three reference transcript sequences with Bowtie2 [27]. The *reads mapping rate* is the ratio of reads that are aligned to the annotated transcripts sequences. This ratio measures the sensitivity that the annotations capture the expressed mRNA sequences. As shown in Table 2, the *mapping rates* for GASS and Ensembl-rheMac2 are very close on the 10,096 common genes.

**Table 2:** **Description of rhesus RNA-Seq datasets for GASS evaluation**

**Accession number in NCBI SRA**	**Tissue**	**Read number**	**Read length**	**Reads mapping rate (%)**		
				**Ensembl-rheMac2 common genes**	**GASS common genes**	**RefSeq-rheMac3 whole gene set**
SRR299127^1^	Liver [28]	15,320,560	76 bp	11.19	11.94	8.94
SRR832953^1^	Brain hippocampus [29]	70,346,332	100 bp	27.89	26.89	22.07
SRR594464^2^	Brain [30]	26,487,487	80 bp	17.97	16,20	15.21

^1^The datasets are single-ended RNA-Seq data.

^2^The dataset is paired-end RNA-Seq data.

The alignment results were incorporated into GAAP pipeline to assess the specificity of annotation at the gene, transcript, exon, and splicing junction levels. The results are shown in Tables 3, 4, 5, and 6. Owing to its conservative and accurate annotation, RefSeq-rheMac3 possesses the highest specificity at almost all levels. GASS possesses higher specificity than Ensembl-rheMac2 at gene, transcript and exon levels. GASS produces the most abundant information, but keeps better accurate performance than Ensembl-rheMac2.

**Table 3:** **Evaluations with RNA-Seq dataset at gene level**

**Data source**	**# of annotated genes**			**# of mapped gene**		
	**Ensembl-rheMac2**	**GASS**	**RefSeq-rheMac3**	**Ensembl-rheMac2**	**GASS**	**RefSeq-rheMac3**
SRR299127	10,096	10,096	6,274	5,335(52.85%)	5,979(59.22%)	3,714(59.19%)
SRR832953				9,379(92.90%)	9,496(94.05%)	5,703(90.89%)
SRR594464				8,305(82.27%)	8,376(82.96%)	5.265(83.92%)

**Table 4:** **Evaluations with RNA-Seq dataset at transcript level**

**Data source**	**# of annotated transcripts**			**# of mapped transcript**		
	**Ensembl-rheMac2**	**GASS**	**RefSeq-rheMac3**	**Ensembl-rheMac2**	**GASS**	**RefSeq-rheMac3**
SRR299127	21,054	35,880	6,360	9,464(44.95%)	16,429(45.79%)	3,737(58.75%)
SRR832953				17,793(84.51%)	33,158(92.41%)	5,752(90.44%)
SRR594464				15,945(75.73%)	28,733(80.08%)	5.295(83.25%)

**Table 5:** **Evaluations with RNA-Seq dataset at exon level**

**Data source**	**# of annotated exons**			**# of mapped exon**		
	**Ensembl-rheMac2**	**GASS**	**RefSeq -rheMac3**	**Ensembl-rheMac2**	**GASS**	**RefSeq-rheMac3**
SRR299127	122,454	146,364	45,540	16,696(13.63%)	24,001 (16.40%)	9,341(20.51%)
SRR832953				108,940(88.96%)	131,393(89.77%)	43,398(95.29%)
SRR594464				94,115(76.85%)	102,831(70.25%)	40.291(88.47%)

**Table 6:** **Evaluations with RNA-Seq dataset at splicing junction level**

**Data source**	**# of annotated junctions**			**# of mapped junction**		
	**Ensembl-rheMac2**	**GASS**	**RefSeq -rheMac3**	**Ensembl-rheMac2**	**GASS**	**RefSeq-rheMac3**
SRR299127	103,757	117,577	39,230	3,600(3.47%)	5,887(5.01%)	2,226(5.67%)
SRR832953				82,925(79.92%)	91,386(77.72%)	36,330(92.60%)
SRR594464				59,384(57.23%)	55,517(47.21%)	29.988(76.44%)

**Figure 6:**
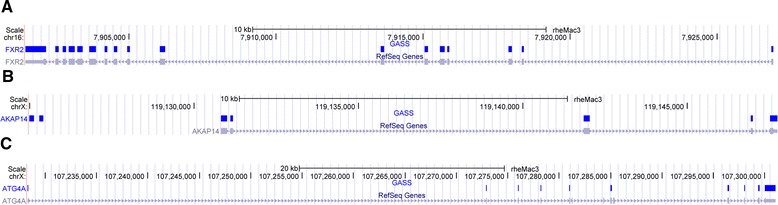
**Three genes annotations from GASS and RefSeq-rheMac3. A)** For gene *FXR2*: the annotations from GASS and RefSeq-rheMac3 are identical. **B)** For gene *AKAP14*: GASS identified two extra exons beyond the RefSeq-rheMac3 annotation. **C)** For gene *ATG4A*: GASS missed three exons in the RefSeq-rheMac3 annotation.

Figure 6 shows some detail transcripts annotation from GASS and RefSeq-rheMac3. As shown in Figure 6(A), for gene *FXR2* in *chromosome 16*, GASS produces identical annotation with RefSeq-rheMac3. As shown in Figure 6(B), for gene *APAK14* in *chromosome X*, GASS identifies two extra exons beyond RefSeq-rheMac3 annotation. As shown in Figure 6(C), for gene *ATG4A* in *chromosome X*, three exons annotated by RefSeq-rheMac3 are missed in GASS annotation.

### Mis-assembly of rheMac3 genome leads to mis-annotations in RefSeq-rheMac3

**Figure 7:**
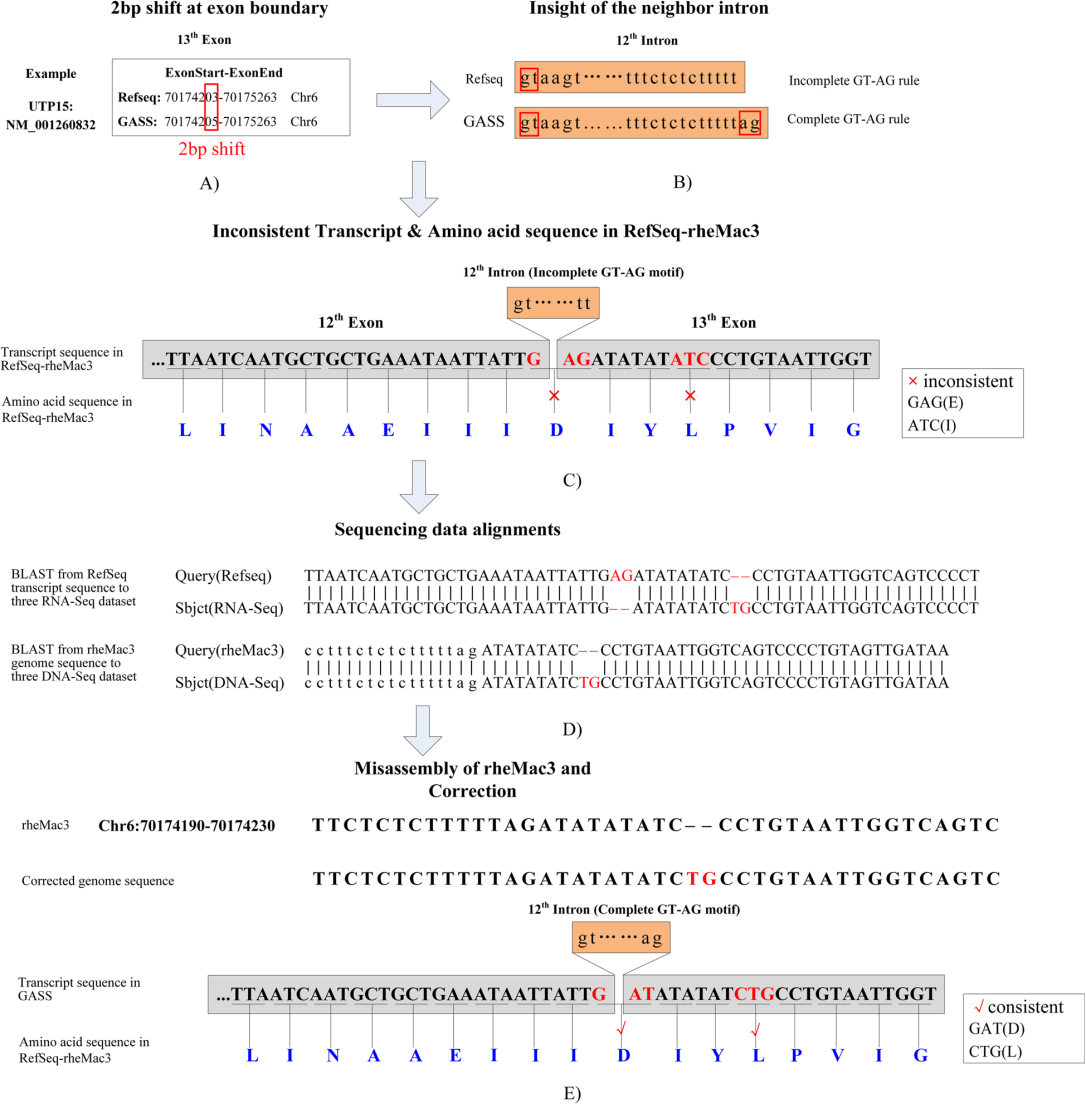
**Mis-annotation for Refseq on rheMac3.** For transcript *NM_001260832*: **A)** The 12^th^ exon has a 2 bp shift between the annotation of RefSeq-rheMac3 and GASS. **B)** The 12^th^ intron cannot meet the “GT-AG” canonical splicing site. **C)** The amino acids coded with triple codon from nucleotides sequence of RefSeq transcript are inconsistent with the amino acid sequences from RefSeq protein database. **D)** Three RNA-Seq datasets and three DNA-Seq datasets are mapped to the sequences from the 12^th^ exon and 13^th^ exon, “AG” cannot be mapped to RNA-Seq datasets and DNA-Seq datasets, meaning that the assembly for the 12^th^ intron is wrong. **E)** The corrected genome sequences and annotations are given, and the transcript nucleotide sequences and the codon amino acid sequences are consistent.

During the comparisons of the three databases, we found more than 2,000 exons with 2 bp shifts on splicing donor or acceptor between GASS and RefSeq-rheMac3. Some of the 2 bp-shift boundaries were checked in detail. As shown in Figure 7(A), for transcript *NM_001260832* of gene *UTP15*, the 12^th^ exon has a 2 bp shift (70174203 vs. 70174205) between the annotation of RefSeq-rheMac3 and GASS. And then we found incomplete GT-AG canonical splicing sites in the 11^th^ intron in Refseq-rheMac3, as shown in Figure 7(B). For Refseq-rheMac3, the first two nucleotides of 11^th^ intron are “gt” while the last two are “tt”, where the “GT-AG” canonical splicing sites misses the “AG”. Meanwhile, as shown in Figure 7(C), the amino acids coded with triple codon from RefSeq nucleotides are inconsistent with the amino acid sequences from RefSeq protein database. For further validation, three RNA-Seq and two DNA-Seq datasets (See Additional file 1: Table S4) were aligned to the Refseq-rheMac3 transcript and rheMac3 genome respectively. As shown in Figure 7(D), the highly consistent alignment results support the following conclusions: ① RNA-Seq alignments prove that the splicing junction should include the complete “GT-AG” canonical splicing sites. ② DNA-Seq alignments prove that the current rheMac3 genome assembly misses two “TG” nucleotides between positions 70174212 and 70174213 of *Chromosome 6*. The corrected genome sequences and annotations are given in Figure 7(E), and the transcript nucleotide sequences and the codon amino acid sequences are consistent. Another example, the analysis of transcript *NM_001260538* is given in Additional file 1: Figure S8.

From the above analysis, we infer that the 2 bp-shifts of exon boundary in RefSeq-rheMac3 are the mis-annotations caused by the mis-assembly of rheMac3 genome. GASS presents the corrected transcript sequences and maintains the complete GT-AG canonical splicing sites.

## Conclusions

This paper proposed GASS, a computational pipeline, to build genome annotations based on species similarity. GASS includes the following three steps: ① Use BLAST to conduct the alignments from annotated species to an un-annotated genome; ② Search the optimal alignment combinations with the shortest path model; ③ Control the annotation quality, and build the annotation database.

For validation, GASS was applied to annotate rhesus genome based on the human annotations. GASS annotations were evaluated with comparing to two existing rhesus structural genome annotation databases (RefSeq-RheMac3 and Ensemb-RheMac2) – directly and with RNA-Seq data. The annotation produced by GASS was evaluated at the gene, transcript, exon and splicing junction levels.

GASS finds most exons, splicing junctions, and transcripts annotated by RefSeq-RheMac3. The sensitivity of GASS is higher than RefSeq-RheMac3 and close to Ensemb-RheMac2. The specificity of GASS is higher than Ensemb-RheMac2 at almost all levels.

Compared with traditional annotating methods, GASS has the following advantages: ① It is easier to use without requiring for extra ESTs, protein sequences, and RNA-Seq data. ② GASS produces eukaryotes genome annotations within several hours. The alignments with BLAST are the most time consuming task and it took about 5 days to align the human annotations to the rhesus genome. Overall, it takes no more than 10 days to produce the annotations for a species with similar size of rhesus genome. ③ Experiments show that GASS produces genome annotations with sufficient abundance and accuracy.

On the other hand, there are also some limitations in this study: ① GASS depends on the annotated information of the similar species. Consequently, GASS misses the information that is specific in the unannotated species. This shortcoming will be improved with RNA-Seq data in our future study. ② GASS provides accurate exon boundary definitions, but it does not identify the boundaries of the coding regions.

Trinity [5] is a tool for the *de novo* full-length transcriptome reconstruction with RNA-Seq dataset. In order to evaluate the overlap of produced annotations when using methods with different data souces, a RNA-Seq data (NCBI SRA SRR594464) was assembled with Trinity. There are 34,068 transcripts were obtained. ① All of the transcripts from Trinity were aligned to GASS’s transcript-sequences with BLAST. There were 49.86% (16,989) Trinity transcripts found in GASS with more than 60% nucleotide identity. ② Conversely, all of the transcripts from GASS were aligned to Trinity’s transcript-sequences with BLAST. There were 45.23% (27,474) GASS transcripts found in Trinity with more than 60% nucleotide identity. ③ Moreover, there were 69.7%, 34.81% and 30.08% reads that can be aligned to rhesus genome, Human-GencodeV14 and Ensembl-rheMac2 with 3 bp mismatches respectively. Therefore, the overlap between GASS and trinity is reasonable considering the *reads mapping rates*.

Genome annotation builds the foundation for interpreting the laws of life genetics. With insight into genome, researchers can plan experiments, speculate the function of a gene product, predict the loci of genes, and conduct follow-up studies and analysis. GASS offers a quick view of genome structural annotation with sufficient accuracy and abundance for an un-annotated genome expressly. The required information is the annotations of a similar species and the genome sequences for the un-annotated species. With its inherent characteristics, GASS could be very useful for some specific situations.

### Availability of supporting data and source codes

The GASS source codes and the produced rhesus annotations are available at http://gassflow.codeplex.com/.

The three RNA-Seq datasets to evaluate GASS, RefSeq-rheMac3 and Ensembl-rheMac2 on subsection “Evaluation with RNA-Seq datasets” are from NCBI SRA, whose accession number are SRR299127 [28], SRR832953 [29] and SRR594464 [30] respectively. The detail descriptions of the datasets are given at Table 2.

The three RNA-Seq and two DNA-Seq datasets to prove the mis-assembly of rheMac3 genome on subsection “Mis-assembly of rheMac3 genome leads to mis-annotations in RefSeq-rheMac3” are from NCBI SRA. The accession numbers for RNA-Seq datasets are SRX424026 [31], SRX209571 [32] and SRX518478 [33]; and the accession number for DNA-Seq datasets are SRX489030 [34], SRX480828 [34].

## Additional file

Additional file 1:
**The file gives the details of some evidences to support our study and some figures and tables mentioned in the main manuscript file.**
