# Supplementary material for: GASS: genome structural annotation for Eukaryotes based on species similarity
Source: BMC Genomics. 2015 Mar 4;16(1):150. doi: 10.1186/s12864-015-1353-3 (PMC4352269; doi:10.1186/s12864-015-1353-3)
Supplement: Additional file 1: — The file gives the details of some evidences to support our study and some figures and tables mentioned in the main manuscript file. [file 12864_2015_1353_MOESM1_ESM.docx]

# Supplementary Data

1. BLAST running command and the parameters

The running command of the BLAST running in our study is as follows:

>blastn -query $file1 -db $index_file -out $out_name -task blastn -dust no -outfmt 6 - num_threads 16 -evalue 0.1 -max_target_seqs 3

①‘*max_target_seqs’*, which controls the number of reported aligned targets, is set to 3.

②The *outfmt,* which permits formatting fields from the BLAST tabular format, is set to 7.

③The *evalue* threshold is set to 0.1.

1. Supplementary Figures

Figure S1. Alignment from query sequences to subject sequences. The query sequences are the exon-sequences of the annotated species. The subject sequences are the genome sequences of the un-annotated species. According to BLAST alignment results, one exon-sequence is possible to be mapped to several locations of same chromosome or different chromosomes. As shown the exon1in the figure S1 means exon1 can be mapped to two positions in chr1 and one position in chr2.


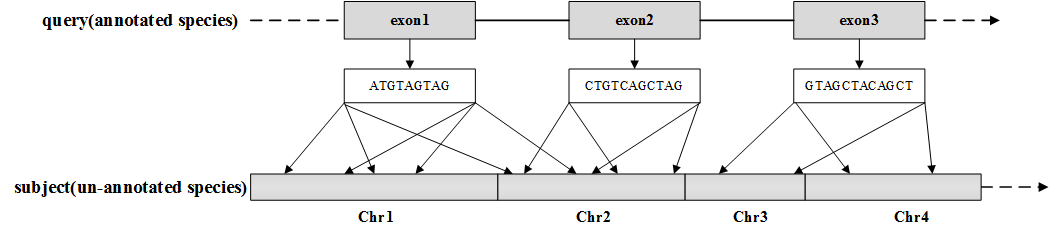


Figure S2. Checking the canonical splicing sites and integrate the transcripts annotations. For eukaryotes, 98.71% of the splicing junctions contain a canonical splicing site, “GT-AG”. GASS checks splice sites with conservative GT and AG dinucleotide. As shown the exon_1 in the figure, when GASS cuts the exon_1’s sequences from the genome sequences, the exon_1’s splicing boundary must meet GT and AG dinucleotide.


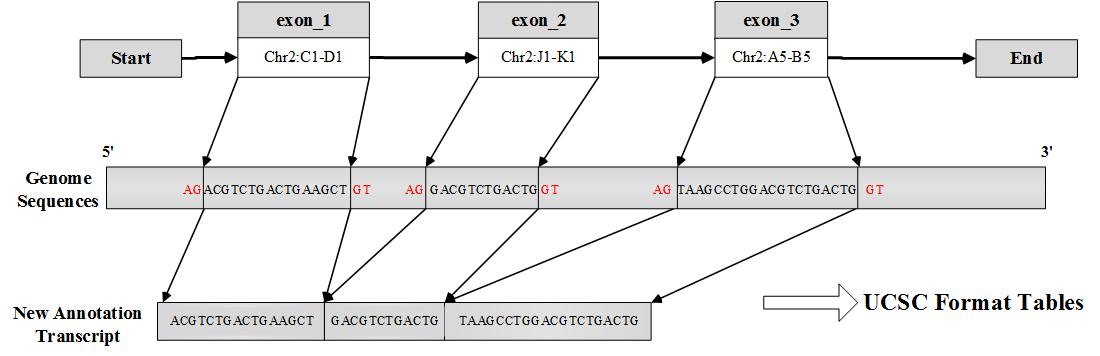


**Algorithm.**

The pseudo code of GASS algorithm

**Input:** The file of BLAST results

The GTF file of annotation for known species

The reference chromosome or no reference chromosome

The genome sequence of un-annotated species

**Output:** Sequence formal of un-annotated species

Table formal of un-annotated species

**For** i-1 to **n-1** do: // n is the number of exon in current transcript

**For** j-1 to **M_i_** do: // M_i_ is the number of mapping positions for$i^{th}$exon of current transcript

**For** k-1 to **M_i+1_** do: // M_i+1_ is the count of mapping positions for$(i+1)^{th}$exon

if $chr_{i+1}!=skip$ and $chr_{i}!=skip$:

if $chr_{i+1}==chr_{i}$:

$$chr\_dis\tan ce_{i,i+1}=0$$

if position consistency:

$$Strand\_direction_{i,i+1}=0$$

$$d_{i,i+1}=chr\_dis\tan ce_{i,i+1}+Aligement_{i,i+1}+Strand\_direction_{i,i+1}$$

else ;

$$d_{i,i+1}=+\infty$$

else:

$$d_{i,i+1}=+\infty$$

end

else:

$$d_{i,i+1}=skip\_identity$$

$$f_{i+1}=\min(d_{i,i+1}+f_{i})$$

end

end

end

end

**return** (optimal exon combination)

**for** each exon in optimal exon combination:

if strand=+:

if splice site is GT-AG:

cut sequence from un-annotation species genome

end

end

if strand =-:

if splice site is CT-AC:

cut sequence from un-annotation species genome

get the complementary sequences

end

end

End

Figure S3. Pseudo code of GASS. Pseudo code of GASS, which is coded in Python. Firstly, exons sequences of annotated species are mapped to un-annotated species with BLAST. Then, GASS takes the alignments results from BLAST as an input, in this stage, we must also provide an annotation file for annotated species in GTF format. A key point in the pipeline is the shortest path model. One merges alignment position and quality, distance between two neighbor exons, canonical splicing site, and gene strand direction in the one-cost. Last cut the exons sequences based on the positions and integrate the exons sequences as the format in UCSC.

Figure S4. Transcript B is the subset of transcript A. the solid lines means the two exons have identical start and end points.


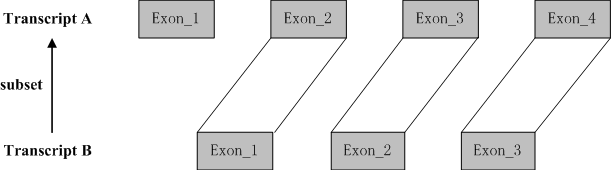


Figure S5. Common genes for RefSeq-rheMac2 and Ensembl-rheMac2. One choses the common genes based on the name of the genes in RefSeq-rheMac2 and Ensembl-rheMac2. From the figure, Ensembl-rheMac2 only can find 40% of genes that annotated by RefSeq-rheMac2, the percentage shows that the overlap for the two databases is low.


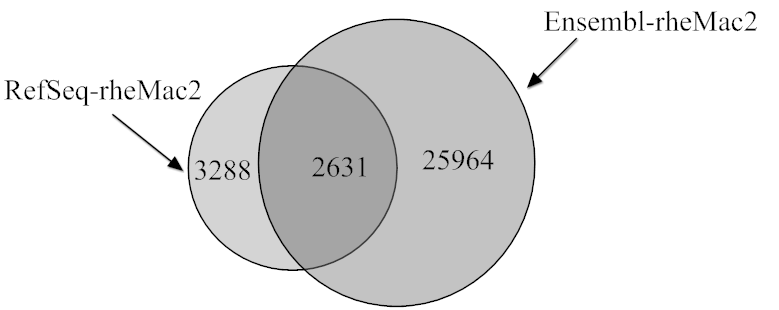

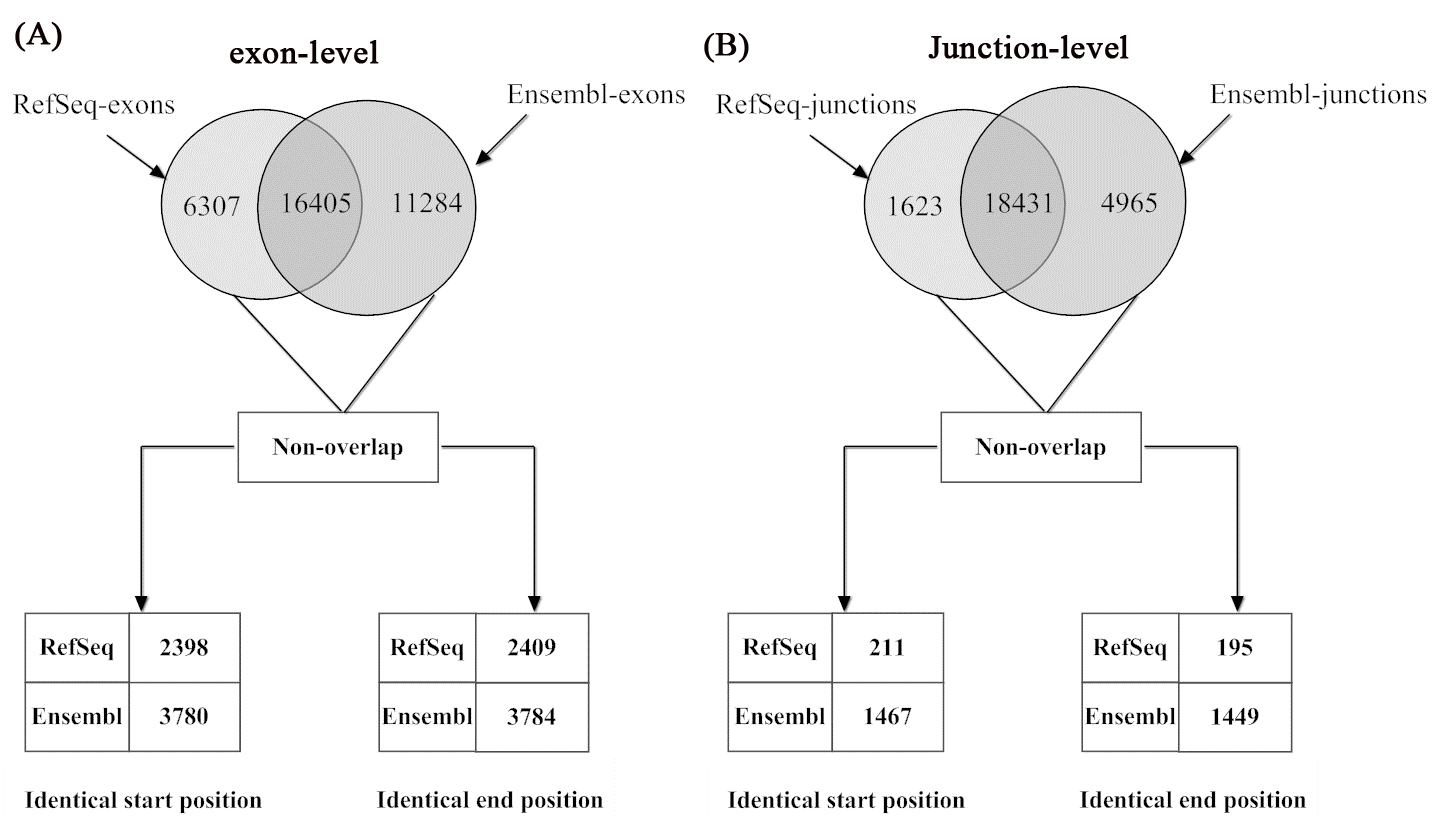


Figure S6. Exon and junction levels for RefSeq-rheMac2 and Ensembl-rheMac2. Figure S6(A). The relationship of exons between RefSeq-rheMac2 and Ensembl-rheMac2. The overlap of the two sets is the common exon. Non-overlap has two parts: one is the exon that has identical end position, the other has identical start position. At exon level, 72.2% of RefSeq-rheMac2 exons are covered by the Ensembl-rheMas2 exons and 21.1% of RefSeq-rheMac2 exons share common starting or end points with Ensembl-rheMac2’s exons. Figure S6(B). The relationship of splicing junction between RefSeq-rheMac2 and Ensembl-rheMac2. The overlap of the two sets is the common splicing junction. Non-overlap also has two parts: one is the splicing junctions that have identical end position, the other splicing junctions that have identical end position. At junction level, 91.9% of RefSeq-rheMac2 splicing junctions are found in those of Ensembl-rheMac2 and 2.02% of RefSeq-rheMac2 splicing junctions share common splicing junction starting or end points with Ensembl-rheMac2’s splicing junctions.


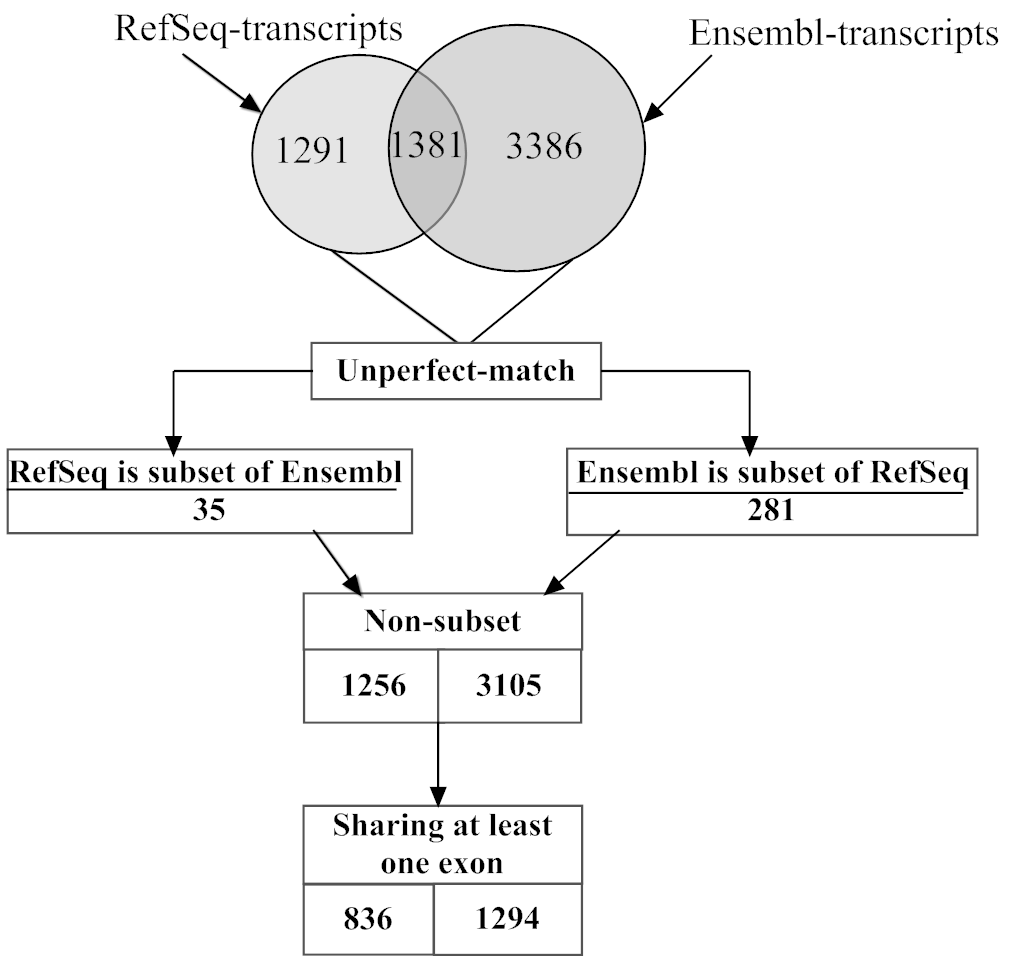


Figure S7. Transcript level compared with RefSeq-rheMac2 and Ensembl-rheMac2. The relationship of transcript between RefSeq-rheMac2 and Ensembl-rheMac2. The overlap of the two sets is the perfect match transcripts. Unperfected match has two parts: one is RefSeq-rheMac2’s transcripts are subset of that Ensembl-rheMac2, the other is Ensembl-rheMac2’s transcripts are subset of that RefSeq-rheMac2. At transcript level, about 50% of the RefSeq-rheMac2 transcripts are covered by the Ensembl-rheMac2 transcripts and 66.56% of RefSeq-rheMac2 transcripts share at least one exon with transcripts in Ensembl-rheMac2.


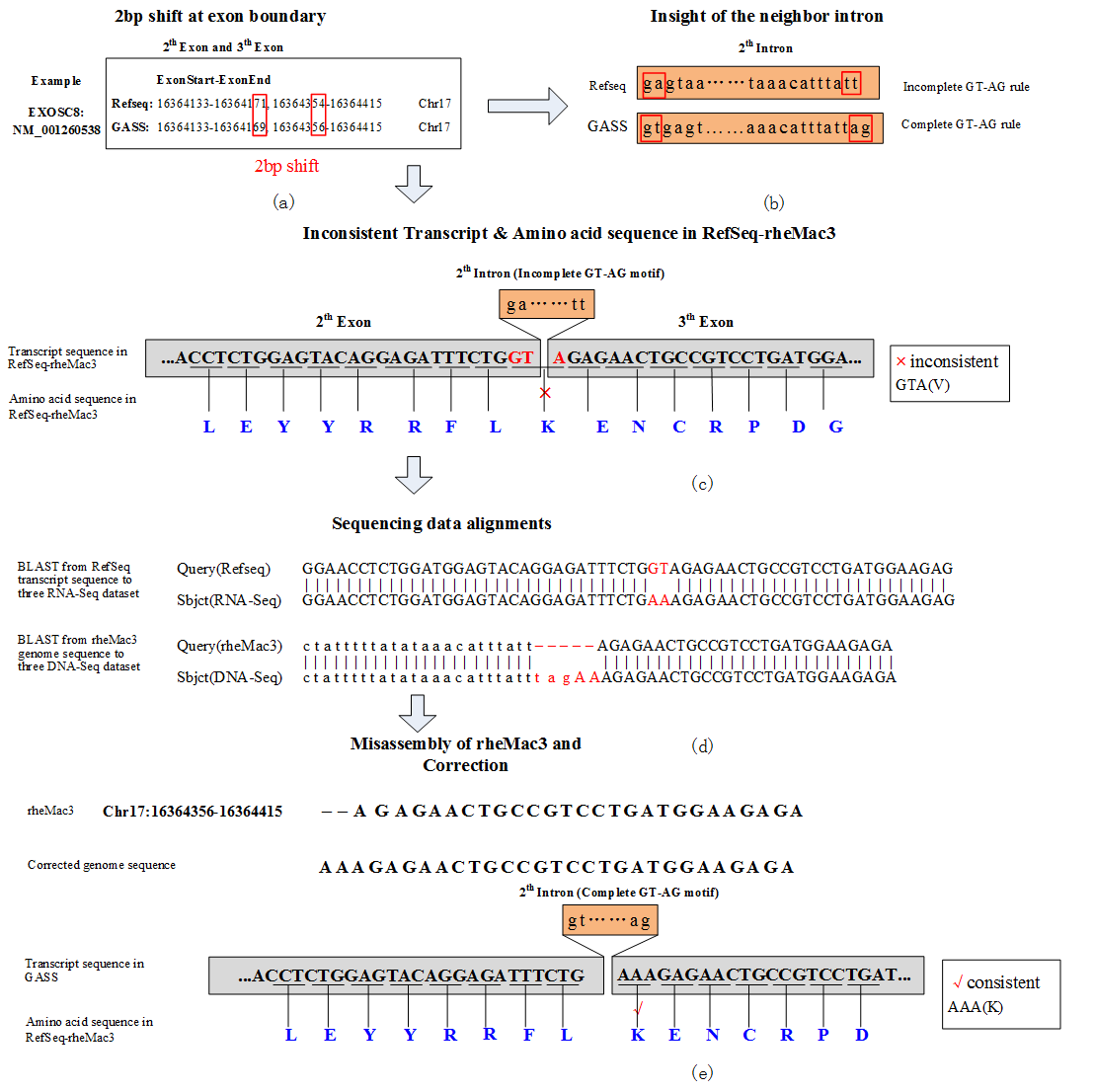


**Figure S8. Mis-annotation of transcript *NM_001260538* for RefSeq on rheMac3.** For the 2th intron of the NM_001260538 cannot meet the canonical splicing site. Furthermore, the amino acid of the junction between the 2th exon and 3th exon is inconsistent with the amino acid sequences provided by RefSeq-rheMac3 itself. Finally, at the sequencing data alignments phase, three RNA-Seq datasets and three DNA-Seq datasets are mapped to the sequences from the 2th exon and 3th exon, ‘GT’ cannot be mapped to RNA-Seq datasets, so‘GT’ should be ‘AA’. From the insight of DNA-Seq datasets, ‘tag’ in the 2th intron cannot be mapped, that means 2th intron in the RefSeq-rheMac3 misses ‘tag’ in the 3’ end of the 2th intron.

1. Supplementary Tables

**Table S1. Summary statistics of GASS and RefSeq-rheMac3 based on common genes.**

| Items | Genes | Transcripts | Exons | Junctions |
| --- | --- | --- | --- | --- |
| RefSeq-rheMac3 | 3,647 | 3,776 | 28,108 | 24,354 |
| GASS | 3,647 | 13,044 | 44,286 | 33,146 |

Based on the 3,647 common genes, RefSeq_rheMac3 has 3,776 transcripts, 28,108 exons and 24,354 junctions. Then, GASS has 13,044 transcripts, 44,286 exons and 33,146 junctions.

**Table S2. Summary statistics of Ensembl-rheMac2 and RefSeq-rheMac2 based on common genes.**

| Items | Genes | Transcripts | Exons | Junctions |
| --- | --- | --- | --- | --- |
| Ensembl-rheMac2 | 2,631 | 4,767 | 27,689 | 23,396 |
| RefSeq-rheMac2 | 2,631 | 2,672 | 22,712 | 20,054 |

Based on the 2,631 common genes, Ensembl-rheMac2 has 4,767 transcripts, 27,689 exons and 23,396 junctions. Then, RefSeq-rheMac2 has 2,672 transcripts, 22,712 exons and 20,054 junctions.

**Table S3. Summary statistics of Ensembl-rheMac2 and GASS based on common genes.**

| Items | Genes | Transcripts | Exons | Junctions |
| --- | --- | --- | --- | --- |
| Ensembl-rheMac2 | 10,096 | 21,054 | 122,454 | 103,757 |
| GASS | 10,096 | 35,880 | 146,364 | 117,577 |

Based on the 10,096 common genes, Ensembl-rheMac2 has 21,054 transcripts, 122,454 exons and 103,757 junctions. Then, GASS has 35,880 transcripts, 146,364 exons and 117,577junctions.

**Table S4. The ID numbers for RNA-Seq and DNA-Seq in NCBI SRA.**

| RNA-Seq ID in NCBI SRA | DNA-Seq ID in NCBI SRA |
| --- | --- |
| SRX424026[[1](#_ENREF_1)] | SRX489030[[2](#_ENREF_2)] |
| SRX209571[[3](#_ENREF_3)] | SRX480828[[2](#_ENREF_2)] |
| SRX518478[[4](#_ENREF_4)] |  |

1. Zhang X, QF Y, HB W, Y Z: **Species-specific alternative splicing leads to unique expression of sno-lncRNAs**. *BMC Genomics* 2014, **15**(287).

2. Zhang S, Liu C, Yu P, Zhong X, Chen J, Yang X, Peng J, Yan S, Wang C, Zhu X *et al*: **Evolutionary interrogation of human biology in well-annotated genomic framework of rhesus macaque**. *Molecular Biology and Evolution* 2014, **31**(5):1309-1324.

3. Chen J, Peng Z, Zhang R, Yang X: **RNA editome in rhesus macaque shaped by purifying selection**. *PLoS Genet* 2014, **10**(4):e1004274.

4. Barrenas F, Palermo R, Agricola B, MB A: **Deep transcriptional sequencing of mucosal challenge compartment from rhesus macaques acutely infected with simian immunodeficiency virus implicates loss of cell adhesion preceding immune activation**. *J Virol* 2014, **88**(14):7962-7972.
